# Reducing Smoking Among People With Schizophrenia: Perspectives on Priorities for Advancing Research

**DOI:** 10.3389/fpsyt.2018.00711

**Published:** 2018-12-18

**Authors:** Amanda L. Baker, Debbie Robson, Sharon Lawn, Marc L. Steinberg, Sandra Bucci, Ann McNeill, David J. Castle, Billie Bonevski

**Affiliations:** ^1^School of Medicine and Public Health, University of Newcastle Newcastle, NSW, Australia; ^2^Institute of Psychiatry, Psychology and Neuroscience King's College London, London, United Kingdom; ^3^Flinders Human Behaviour and Health Research Unit, Department of Psychiatry, Margaret Tobin Centre, College of Medicine & Public Health, Flinders University Adelaide, SA, Australia; ^4^Division of Addiction Psychiatry, Rutgers Robert Wood Johnson Medical School New Brunswick, NJ, United States; ^5^Division of Psychology and Mental Health, School of Health Sciences, Faculty of Biology, Medicine and Health, Manchester Academic Health Science Centre University of Manchester, Manchester, United Kingdom; ^6^Greater Manchester Mental Health NHS Foundation Trust Manchester, United Kingdom; ^7^Department of Psychiatry, University of Melbourne Melbourne, VIC, Australia; ^8^Department of Psychiatry, St Vincent's Hospital Melbourne Fitzroy, VIC, Australia

**Keywords:** smoking, smoking cessation, schizophrenia, research, health priorities, severe mental illness, mentally Ill persons, vulnerable population

## Abstract

Although tobacco smoking is very common among people with schizophrenia and has devastating effects on health, strategies to ameliorate the risk are lacking. Some studies have reported promising results yet quit rates are much lower than in the general population. There is a need to advance research into smoking cessation efforts among people with schizophrenia. We posed the following question to five leading international experts in the field: “What are the top three research ideas we need to prioritize in order to advance the field of reducing smoking amongst people with schizophrenia?” They identified three broad priorities: (i) deeper understanding about the relationship between smoking, smoking cessation and symptomatology; (ii) targeted, adaptive and responsive behavioral interventions evaluated with smarter methodologies; and (iii) improvements in delivery of interventions. Efforts should be made to establish a collaborative international research agenda.

## Introduction

We live in an era when tobacco, which is one of the most harmful substances for human health, is legal and widely available in combustible form. Whilst tobacco control strategies have greatly reduced smoking rates among the general population in high income countries, smoking rates among people with schizophrenia remain very high [e.g., (1, 2)]. Schizophrenia is often complicated by physical comorbidities and substance use (1). That well over half of people worldwide with a diagnosis of schizophrenia smoke, should be (1) of great concern (given our knowledge of the effects of smoking on quality of life, morbidity and mortality) and (2) a dynamic area of research (given the often intractable nature of schizophrenia and what its relationship with smoking might tell us). Sadly, neither of these are true and up until recently, little attention was paid to this issue. We (AB, BB and DC) asked leading researchers from Australia, the UK and the USA the following question, “What are the top three research ideas we need to prioritize in order to advance the field of reducing smoking amongst people with schizophrenia?” Their views represent opinion rather than systematic review, and are provided below, followed by our commentary.

## The Perspective of a Social Work Clinician and Mental Health Carer (Australia)

Professor Sharon Lawn's top three research ideas are drawn from her experiences in the past 20 years of undertaking ethnographic research, being a community-based clinician observing (at close quarters and over extended periods) the lives of people with schizophrenia, and from living with a smoker with schizophrenia.

### Understanding More About the Overlap Between Symptoms of Schizophrenia and Withdrawal

We need to understand more about how the experience of schizophrenia and the experience of nicotine dependence and withdrawal interact with each other, at the basic level of “the science” right through to the person's day-to-day attempts to manage the two. Many of the interventions proposed focus on mental illness symptoms and nicotine dependence/withdrawal as separate processes rather than investigating how the person navigates the two at the levels of symptoms, pharmacotherapy and subjective experience. I flagged this need a long time ago (3), calling for the integration of care to recognize and treat psychosis and nicotine withdrawal together. The problem is that research, and then clinical practice, has resorted to offering standard quit strategies and nicotine replacement therapy (NRT) use. Outcomes of these interventions have lagged for smokers with schizophrenia and sustaining quit status has been elusive. People with schizophrenia tend to relapse quickly to smoking because schizophrenia is a condition that is lived with every day, is changeable and complex to manage and—in the absence of support that addresses symptoms and dependence together—reaching for a cigarette becomes the default option for coping.

### Prevention and Early Intervention

Once the person becomes a smoker, a complex interplay of need, coping, psychological, and physiological interactions between their mental health and their smoking is established and becomes insidiously reinforced. Also, from the carer's perspective, there is overwhelming frustration and sadness as the smoker ages prematurely, trying to battle multimorbidity, and dying before their time. Therefore, research must focus on prevention and early intervention for people at risk and those with emerging psychosis, so that they never become smokers in the first place, or that they quit early.

### Targeted Pharmacotherapies and Practical Quit Strategies

Finally, we need more efficacious smoking cessation interventions that take account of the knowledge gained from my first identified priority (above). This includes pharmacological treatments that are safe and effective, as well as interventions that provide real support to the person “in the moment,” not just sending them away with NRT and brief counseling. Interventions that involve quit strategies that are predominantly “abstract” (e.g., weighing up the pros and cons, planning and identifying a range of strategies) have limits for many people with schizophrenia and are likely to be completely useless in the moments of escalating stress and distress when the person is unable to “call to mind” the reasoning that underlies such abstract quit strategies. It may in fact escalate the person's anxiety, demanding clarity of thinking when thinking through options is at its most difficult. Additionally, repeated failure reinforces feelings of hopelessness. This is why apps that help address “the moments” in the “here and now” without the need to work out how to operationalise abstract concepts, are also worth further research.

## The perspective of a Clinical Psychologist / Co-director of a Complex Trauma and Resilience Research Unit, Greater Manchester Mental Health NHS Foundation Trust (UK)

Dr. Sandra Bucci's top three ideas are drawn from her clinical and research experience focusing on psychological treatments of people diagnosed with schizophrenia, those in the early stage of psychosis and young people at ultra-high risk of psychosis.

### Research Needs to Acknowledge Clinical Complexity and Treatment Should Aim for Realistic Goals Chosen by Patients

Asking people with a severe mental health problem to give up or reduce smoking is complex. Researchers more often than not argue that the ultimate goal for someone with a diagnosis of schizophrenia should be cessation (4). However, motivational problems, self-medication, being part of a social group, the physiological effects associated with nicotine intake, and health and social inequalities apparent in people with severe mental health problems are just a few of the factors that influence the complex interaction between smoking and mental health. It may therefore be preferable to encourage patients to set realistic goals, working toward cessation.

### Providing Digital Technologies to Impact on Daily Living and Newer Methodologies

Many programs target smoking behaviors in people with severe mental health problems, with varied outcomes. We can provide smokers all the information they need regarding the negative health effects of smoking. We can also attempt to replace smoking behaviors with other, more adaptive strategies during times of stress. However, these approaches in and of themselves are limited as they do not impact people in-the-moment, at the time the smoker experiences a craving and is in most need of support. There is a mismatch between the rather static nature of providing support for smoking cessation to people who often find it difficult to resist cravings and urges to smoke, and stressors that are momentary and contextual (5). This is where the digital revolution, primarily through the availability of smartphones and smartphone apps, may help. We must leverage the opportunities digital technology afford, by developing intervention packages that can be delivered in the moment, in the context in which stressors occur. Digital technologies provide an unprecedented opportunity to reach people in a timely manner in the context of their daily life. There is a narrowing gap in smartphone ownership in individuals with a diagnosis of schizophrenia (6), highlighting the potential for healthcare programs targeting smoking behavior to be taken from the clinic to people's personal environment, unconstrained by location and time.

Methodologies such as Just In Time Adaptive Interventions (JITAI) that use digital technology as the modality for intervention delivery, may be the optimal platform to provide timely, contextual, in-the-moment support to people who find it difficult to recall or use treatment strategies during stressful moments where pressures on cognitive load and resources are most intense (5). JITAI can provide the right type and amount of support, at the right time by adapting to the individual's changing internal and contextual state (7). This approach is particularly well-suited to delivering smoking cessation/reduction programs among people with schizophrenia, affording us the opportunity to prompt and nudge people at the time they are most vulnerable.

Developing evidence-based interventions that are rapidly available and accessible at the population level are a priority. Historically, researchers have relied on using randomized controlled trials (RCTs) to explore the effectiveness of smoking cessation/intervention programs. However, RCTs are time consuming and do not in fact tell us which aspects of the intervention are effective. In standard RCTs, the intervention is typically fixed at trial onset and does not evolve over the course of the trial. As we move toward using digital technologies to nudge and prompt people regarding smoking behaviors in-the-moment, we run the risk that the technology is outdated or even obsolete at the end of the trial period. Adaptive approaches to clinical trials should explore the implementation of more rapid trial designs to ensure effective interventions are available in a timely and accessible way (8).

### Staff Attitudes

Thirdly, to ensure effective dissemination of effective programs, we need more research into how best to change staff attitudes to smoking cessation in mental health settings. Staff can be reticent to encourage people with severe mental health problems to quit smoking. The success of smoking cessation programs is influenced not by patient uptake, but also clinician views and attitudes about smoking behaviors.

## The Perspective of Researchers in Tobacco Addiction (UK)

In their perspective, Dr. Debbie Robson combines experience in mental health nursing and research in tobacco addiction with that of Professor Ann McNeill, who has led numerous research and policy initiatives to reduce smoking among those with mental illness.

### We Don't Know Enough About the Relationship Between Smoking and Schizophrenia

Why is the relationship so strong? Evidence has been found for shared familial and genetic risk factors, limited evidence for schizophrenia causing people to smoke, and limited evidence for smoking causing schizophrenia (9, 10). Research is hampered by poor routine surveillance of smoking prevalence, in contrast to internationally agreed robust measures used to track smoking in general populations. There is a paucity of large longitudinal studies exploring causal mechanisms, assessing frequency/heaviness of smoking, nicotine intake, quitting behaviors, and detailed objective measurement of the mental illness and their interactions.

### We Have Failed to Identify Appropriate Ways Out of Tobacco Addiction for People With Schizophrenia

Tobacco control and smoking cessation interventions are usually derived/adapted from evidence generated in general population samples (11). Very few are co-designed by smokers with schizophrenia. Although the outcomes from using existing evidence-based treatments for smoking cessation could be improved by finding ways to promote better adherence, people with schizophrenia deserve investment to develop bespoke interventions tailored to their illness-related psychological, cognitive, and social needs (12). For example, we should ascertain what outcomes smokers with schizophrenia value most and what treatments are acceptable. We then need to co-design interventions with people with schizophrenia and key stakeholders.

### A Lack of Engagement of the Health Workforce

People with lived experience of schizophrenia have more frequent contact with health services (13) and a visit to a hospital inpatient/outpatient setting should be an opportune time to promote key messages about smoking and offers of support to quit. This is undermined by poor knowledge and therapeutic nihilism (14) among health professionals and resistance to implementing comprehensive smokefree policies (15). Improving the capability of the health workforce so that every clinician is committed to and has the competence to initiate conversations and support those with lived experience of schizophrenia to quit smoking, is vital. We need to develop and evaluate novel ways to integrate smoking and tobacco dependence treatment, education and training into routine healthcare.

## The Perspective of a Clinical Psychology Researcher With a Special Interest in Enhancing Motivation to Change (USA)

Dr. Marc Steinberg (16–23) has a long-standing interest in researching smoking cessation treatments among people from socially disadvantaged backgrounds, including people with schizophrenia.

### Evaluating How to Best Address Reduced Distress Tolerance/Task Persistence in This Population

Given low abstinence rates, we should consider additional behavioral supports to combine with empirically supported pharmacological approaches. Smokers with schizophrenia have reduced task persistence/distress tolerance as compared to smokers without psychiatric comorbidity (16–18), and this may be a fertile target for counseling. Approaches such as traditional cognitive behavior therapy (CBT) focusing on thoughts related to persistence or distress tolerance, and Acceptance and Commitment Therapy (ACT) focusing on increasing psychological flexibility should be examined in this population. As Baker (19) recently suggested, the field should consider factorial designs to determine optimal counseling components for people with schizophrenia, and to empirically test whether various intervention combinations are more or less effective.

### Examining Motivational Interviewing Interventions

While smokers with schizophrenia report being as interested in quitting as their peers without psychiatric comorbidity (20), the field needs to do better in increasing the number of quit attempts, and, importantly, increasing the number of appropriately *aided* quit attempts. An empirically validated approach likely to be useful in this endeavor is Motivational Interviewing (MI) (21). Adaptations of MI can support quit attempts as well as tobacco dependence treatment seeking in smokers with schizophrenia (21, 22). The literature on MI for smokers with schizophrenia is sparse, however, with many important questions remaining unanswered. Future studies should examine MI not only for motivating quit attempts, but also enhancing such attempts.

### Training Providers to Address Tobacco Use

Finally, the field should strive to increase the number of quit attempts by encouraging the healthcare system to address tobacco use in their patients. In addition to providing and evaluating continuing education opportunities, we must increase the number of graduate programs and medical schools that include tobacco dependence treatment in their curricula because preliminary evidence suggests that training behavioral healthcare providers improves the chances of their addressing tobacco in their patients (23).

## Discussion

Three broad areas were highlighted as priorities for research by our invited experts. The contributions by Lawn and also by Robson and McNeill call for more understanding about the relationship between smoking, smoking cessation and symptomatology, ranging from large longitudinal datasets to monitoring interactions between smoking and symptoms. Intriguingly, two smoking cessation studies among people with schizophrenia recently conducted by the current authors (24–26), in the US and Australia, have found that face-to-face or telephone-delivered interventions with core components consisting of monitoring psychiatric symptoms and understanding medication interactions with tobacco smoking appear promising. In this issue, we^1^ describe the “Quitlink” randomized controlled trial, in which such components will be delivered via quitline.

The second major area for further research was in the area of behavioral interventions. Better preventive measures, early interventions and motivational strategies tailored for people with schizophrenia were seen as important by Lawn and Steinberg. Bucci and Steinberg highlighted the complexity of presentations that take into account specific aspects of schizophrenia that are likely targets of intervention (e.g., distress tolerance and also flexible goals toward smoking cessation). Lawn and Bucci argue strongly for more research into interventions which can be used by smokers with schizophrenia in everyday settings, to strengthen the likelihood of them being able to address smoking in challenging situations. Bucci promotes digital interventions but Lawn also notes the need for smarter medications which could address both mental health and smoking. Bucci and Steinberg both point to the need for new and emerging behavioral interventions to require better and faster methodologies, supporting identification of effective key components and faster dissemination and adaptation to individual needs. Importantly, co-design was noted by Robson and McNeill as likely to yield vital information about valued outcomes and interventions.

Thirdly, all experts lamented the paucity of smoking cessation care provided by health systems and staff. Shifting health care systems and culture, building the capacity and confidence of clinical staff to address smoking and implementing smoke free policies are challenging changes within organizations. Emerging research however suggests that systems and organizational change interventions may provide a sustainable approach to integrating smoking cessation support in settings that care for people with schizophrenia (27–29).

There are a few notable limitations of this paper. Topics identified related mainly to high-income countries. Also, the utility of existing yet contentious smoking cessation treatments such as electronic cigarettes and varenicline was not explored.

The perspectives presented here provide a clear agenda for further research. National and international research collaborations (e.g., Mental health and smoking partnership^2^) should target these priorities with a view to impacting upon the very high rates of smoking among people with schizophrenia.

## Author Contributions

AB conceived of the idea for this paper. AB, DC, and BB suggested contributors. All authors wrote sections of the manuscript and contributed to manuscript revisions.

### Conflict of Interest Statement

The authors declare that the research was conducted in the absence of any commercial or financial relationships that could be construed as a potential conflict of interest.

## Footnotes

^1^ Baker AL, Borland R, Bonevski B, Segan C, Turner A, Brophy L, et al. “Quitlink” - A randomised controlled trial of peer worker facilitated Quitline support for smokers receiving mental health services: study protocol. (Under review in Frontiers in Psychiatry).

^2^http://smokefreeaction.org.uk/smokefree-nhs/smoking-and-mental-health/
